# The Role of Dendritic Cells in TB and HIV Infection

**DOI:** 10.3390/jcm9082661

**Published:** 2020-08-17

**Authors:** Rachel Abrahem, Emerald Chiang, Joseph Haquang, Amy Nham, Yu-Sam Ting, Vishwanath Venketaraman

**Affiliations:** 1 College of Osteopathic Medicine of the Pacific, Western University of Health Sciences, Pomona, CA 91766, USA; rachel.abrahem@westernu.edu (R.A.); emerald.chiang@westernu.edu (E.C.); joseph.haquang@westernu.edu (J.H.); amy.nham@westernu.edu (A.N.); yusam.ting@westernu.edu (Y.-S.T.); 2 Graduate College of Biomedical Sciences, Western University of Health Sciences, Pomona, CA 91766, USA

**Keywords:** dendritic cells, HIV, TB, Mycobacterium tuberculosis, immune responses

## Abstract

Dendritic cells are the principal antigen-presenting cells (APCs) in the host defense mechanism. An altered dendritic cell response increases the risk of susceptibility of infections, such as *Mycobacterium tuberculosis* (*M. tb*), and the survival of the human immunodeficiency virus (HIV). The altered response of dendritic cells leads to decreased activity of T-helper-1 (Th1), Th2, Regulatory T cells (Tregs), and Th17 cells in tuberculosis (TB) infections due to a diminishment of cytokine release from these APCs, while HIV infection leads to DC maturation, allowing DCs to migrate to lymph nodes and the sub-mucosa where they then transfer HIV to CD4 T cells, although there is controversy around this topic. Increases in the levels of the antioxidant glutathione (GSH) plays a critical role in maintaining dendritic cell redox homeostasis, leading to an adequate immune response with sufficient cytokine release and a subsequent robust immune response. Thus, an understanding of the intricate pathways involved in the dendritic cell response are needed to prevent co-infections and co-morbidities in individuals with TB and HIV.

## 1. Incidence and Epidemiology of Tuberculosis

Tuberculosis (TB) is a respiratory infection that originates from the bacteria *Mycobacterium tuberculosis* (*M. tb*). TB is recognized as one of the top 10 leading infectious killers globally; it is also the most common opportunistic infection and a contributing cause of death for HIV patients. Globally, it is estimated that TB has 10 million incident cases and an estimated 1.9 million deaths [1]. According to the World Health Organization’s Annual Tuberculosis Report in 2019, the highest estimated total incidence of TB was in Southeast Asia, with 4,370,000 incident cases. Africa was second to Southeast Asia with 2,450,000 incident cases. Thus, most cases and deaths are in developing countries. Individuals with other comorbidities and factors that suppress the immune system, such as uncontrolled diabetes, HIV infection, chronic renal failure, and use of immunosuppressive drugs, are at higher risk for contracting primary *M. tb* infection and undergoing reactivation of latent *M. tb* infection [2]. Furthermore, social determinants of health, including poverty, undernutrition, lack of access to anti-retroviral therapy (ART) for HIV, and smoking, lead to a higher *M. tb* disease burden and, thus, concentrate the disease in socio-economically disadvantaged countries [3]. In developing regions, such as Sub-Saharan Africa, limited access to ART increases the susceptibility to HIV and other co-infections most commonly seen with TB and HIV comorbidities [4]. Administration of ART reduces TB incidence by 67% [5], as well as mortality if ART is started early [6].

## 2. Pathophysiology of Tuberculosis

Initial infection with *M. tb* involves the inhalation of aerosolized infectious droplets containing the pathogen, which travel down the respiratory tract to infect the lungs’ alveoli. Thereafter, *M. tb* can travel throughout the body via systematic and lymphatic circulation and infect other organs, such as the brain, kidney, bone, or apex of the lungs. Within 2–8 weeks, macrophages—specialized immune cells—mount an immune response by ingesting and destroying the *M. tb*. However, some of the macrophages aggregate and form a granuloma, an immune barrier that encloses and suppresses the *M. tb*, instead of completely clearing the infection [7]. In these granulomas, mature macrophages fuse to form multinucleated giant cells. Alongside macrophages, cells, such as dendritic cells (DCs), neutrophils, natural killer cells, fibroblasts, CD4 T cells, and cytotoxic CD8 T cells are also recruited to the granuloma via cytokine mediation, leading to further containment of the bacterium [8].

Inside the granuloma, effector responses, along with a lack of nutrients and oxygen, cause *M. tb* to become dormant and remain in a non-replicating state. The contained *M. tb* within a granuloma in the lungs is referred to as latent tuberculosis (LTBI). In immunocompromised individuals, a breakdown of immune responses can result in reactivation of *M. tb* [9]. An immune-compromised state promotes liquefaction of the caseum in the granuloma and bacterial replication, thereby promoting cavity formation and the release of *M. tb* to the exterior during coughing, causing spread of the infection to other parts of the lungs [10]. Active *M. tb* deflects the host defense mechanisms via cord factor, preventing fusion between the phagosome and lysosome and degradation of the bacterium [11].

## 3. Incidence and Epidemiology of HIV

The human immunodeficiency virus (HIV) is a species of Lentivirus belonging to the family of Retroviridae and is the primary infectious agent that is responsible for causing acquired immunodeficiency syndrome (AIDS) [12,13]. The major modes of transmission of HIV include unprotected heterosexual intercourse, men who have sex with men, intravenous/injection drug use, and mother-to-child transmission, with certain methods being main contributions to regional epidemics [14].

According to data from the Joint United Nations Program on HIV/AIDS (UNAIDS), there are approximately 38 million people living with HIV, including adults and children. Since its discovery in 1983, the incidence of HIV infections has been reduced by 40% over the years with 1.7 million people newly infected with HIV in 2019 compared to the peak of 2.8 million people infected in 1998 [15,16]. This decline is likely due to the utilization of antiretroviral therapy (ART), which has dramatically reduced the mortality rate among people living with HIV [14]. The World Health Organization (WHO) defines most-at-risk populations who are disproportionately affected by HIV as men who have sex with men, transgender people, people who inject drugs, and sex workers [17].

## 4. Pathophysiology of HIV

Sexual transmission of HIV is the most common route of HIV-1 acquisition worldwide and is characterized by the progressive depletion of CD4+ T lymphocytes [18]. Cells residing within the epithelial surfaces are the initial targets for HIV after mucosal exposure. For HIV to infect cells, its surface envelope glycoprotein, gp120, must be able to bind to primary receptors CD4 and a co-receptor CXCR4 or CCR5 for viral entry [19]. Once inside the cell, the virus will replicate and will induce a cellular immune response and synthesis of viral proteins. Within the first few weeks of symptomatic primary infection, viremia develops accompanied by symptoms in some patients. During the first few weeks of acute HIV infection there are high levels of replicating virus in the cells of the genital tract and continue to remain elevated for the first 10–12 weeks [20,21]. Accordingly, the highest transmission of HIV to other individuals occurs during this period of early infection [22], especially in those that engage in risky behaviors and men who have sex with men (MSM).

A few weeks after, levels of the virus decline, which is seen to coincide with the development of a cellular immune response. Soon after, secondary phase, also known as asymptomatic period or clinical latency, represents a period of ongoing viral replication that is unrecognized by the immune system. The steady decline seen in the CD4+ T cell population is not well understood but is the result that leads to the progression of AIDS [23].

HIV-2 shares structural, antigenic, and genomic characteristics as HIV-1, although it shows a vastly different pathogenic ability in the human host [24]. HIV-2 has a lower infectivity, longer asymptomatic phase, and slower progression to AIDS than HIV-1 [25]. Like HIV-1, HIV-2 has a surface envelope glycoprotein that binds to co-receptor CD4, but at a lower affinity than HIV-1, possibly contributing to the differences seen between HIV-1 and HIV-2 [24].

## 5. Dendritic Cells

Dendritic Cells (DCs) have a significant role in host immune responses against *M. tb* infection. The mononuclear phagocyte system (MPS) is a class of cells that have the specialized function for processing and presenting antigens to activate lymphocytes [26]. The MPS is comprised of monocytes, macrophages, and DCs [27]. Within this system, DCs are known as the most efficient antigen-presenting cells in the immune system. This antigen-presenting function is a key component for the DCs to link the innate immune system with the adaptive immune response. Since protein antigens cannot be recognized by T cells in their native state, DCs are needed to process the complex protein antigens into peptides and conjugate these antigenic peptides to MHC I or II complexes that can then be recognized by CD8 and CD4 T cells, respectively, thereby activating an appropriate adaptive immune response. DCs perform these functions by using different receptors that can detect pathogen-derived components expressed on the plasma membrane (TLR) or within the cytoplasm (NOD-like receptors) [28]. DCs exist in either an immature or mature form [29]. The immature form is found in peripheral tissues, which are responsible for detecting pathogens or injured host cells. This form is very efficient at performing endocytosis; however, they are equally as inefficient at generating peptide-MHC complexes [28]. In order for the correct immune response to activate, the immature DC must become triggered by pathogens to develop into a mature DC. These mature DCs are now more efficient at producing peptide-MHC complexes as well as allowing these complexes to present at the cell surface [29].

Depending on the nature of the pathogen that is endocytosed, the DC activates varying forms of T cell responses through presenting these peptides on either MHC I or MHC II [29]. MHC I antigenic peptide complex are known to be processed forms of antigens synthesized endogenously such as peptides being presented by virally infected cells. MHC II antigenic peptide complexes are known to be from peptides that are associated with exogenous sources such as peptides from endocytosed bacteria. In addition to producing more peptide-MHC complexes, the mature DCs can now migrate into lymphoid organs, which are T cell rich, to stimulate extremely specific T cell responses [30,31]. Within these T cell rich organs, DCs are also responsible for secreting polarizing cytokines that induce CD4+ T cells to differentiate into various subtypes, which include Th1, Th2, Th17, and regulatory T cells [32].

**Figure 1:**
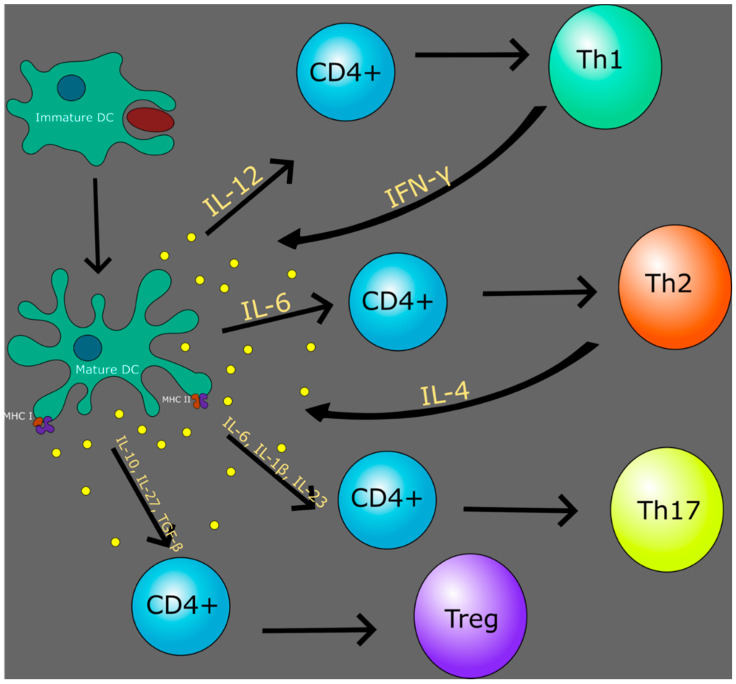
Dendritic Cells (DCs) play a major role in the cytokine response to differentiate various classes of T cells.

When DCs are exposed to an intracellular pathogen, the cell secretes interleukin-12 (IL-12), which differentiates the CD4 T cell into a Th1 cell. Following this, the Th1 cell secretes interferon-gamma (IFN-γ) that causes a positive feedback loop for the DC to further produce IL-12 and produce more Th1 cells [33]. DCs also stimulate Th2 cells in a similar fashion but with the cytokine IL-6, which also leads to a positive feedback loop when the Th2 cell secretes IL-4 to further generate more Th2 cells [33]. Along with Th2 cell activation, IL-6 has been shown to act in a dual role along with IL-1β and IL-23 in activating Th17 cells [34]. This subset is responsible for clearing pathogens as well as inducing tissue inflammation in autoimmune disease [35]. With such a wide range of immune responses, DCs also have a function to prevent autoimmune reactions. This is achieved by inducing the formation and differentiation of Tregs by IL-10, IL-27, and transforming growth factor-β (TGF-β). Tregs are responsible for maintaining self-tolerance and suppressing pathological immune responses by the clonal deletion of self-reactive T cells [36] (Figure 1). Within the MPS, Langerhans cells (LCs) are also an antigen-presenting cell that is present in the epidermis, which is distinctly important for both HIV and TB infections. However, the definition of LCs has been under controversy about whether it is a macrophage or a subset of DC [37]. This stems from the fact that LCs contain characteristics of both macrophages as well as DCs. The macrophage characteristics include the ability to self-renew as well as having a common macrophage precursor from the adult fetal liver [37,38]. The DC characteristics include the ability to migrate into skin-draining lymph nodes as well as stimulating naïve T cells [37]. In identifying the multitude of immune responses that are mediated by DCs, it is clearly evident that in immunocompromised states, an altered DC response will lead to significant negative downstream effects within the immune system.

## 6. HIV and Dendritic Cells

CD4+ T cells are the major targets of HIV infection, although DCs may play a large part as they can influence transmission, target cell infection, and antigen presentation [39]. HIV must overcome the epithelial barrier within the genital tract to reach immune target cells beneath the epithelial lining, where DCs are one of the first immune cells to come in contact with the virus [40,41]. DCs are capable of binding to the viral envelope glycoprotein gp120 present on HIV-1 through high expression of entry receptors C-C chemokine receptor 5 (CCR5), as well as relatively low levels of CD4 [39]. Along with DCs, LCs are also one of the first immune cells that come in contact with the HIV virus. LCs are resistant to HIV infection by binding to the viral envelope glycoprotein gp160 through langerin, which leads to the endocytosis of the virus and prevents infection [42]. However, LCs also express the HIV entry receptors CD4 and CCR5, which mediates the fusion of gp160 and leads to productive infection of the cell [42]. This can lead to transmission and rapid spread of the infection to CD4+ T cells through cell-to-cell interactions [42]. Considering the impact of HIV in DCs compared to LCs, HIV-primed monocyte-derived LCs lead to a higher amount of HIV-specific CD8+ T cells being induced, as well as a lowered induction of Tregs compared to HIV-primed monocyte-derived DCs [43].

However, HIV-1 infection in LCs has been a source of controversy because new studies demonstrate that CD1a+ vaginal dendritic cells (VEDCs) are a more likely possible reservoir and source of HIV-1 transmission in the female genital tract, rather than LCs [44]. Preferential infection of VEDCs is due to their expression of CCR5, the receptor that a majority of new infections by HIV-1 utilizes, as opposed to CXCR4 [45]. VEDCs also lack Birbeck granules, which LCs possess. These granules provide protection from HIV-1 infection. This notion was corroborated by the presence of significant amounts of HIV-1 DNA copies in VEDCs in samples of vaginal tissue from HIV-1 infected women. HIV-1 DNA copies have not been measured before in LCs in vivo. It has also been shown that epidermal dendritic cells that are CD11c+ and enriched in anogenital tissues are more likely to be infected by HIV-1 and efficient at transmitting HIV-1 to CD4+ T-cells. CD11c+ DCs express higher levels of CCR5 and were found to have higher levels of HIV RNA when compared to LCs [46].

DC subsets express other receptors that can bind the envelope glycoprotein gp120, such as which express the C-type Lectin Receptor (CLR) Langerin (CD207) and conventional DC (cDC) express DC immunoreceptor (DCIR). In addition, other CLRs such as DC-specific intercellular adhesion molecule-grabbing non-integrin (DC-SIGN) and dermal DC expressing mannose receptors can also bind the glycosylated gp120 [39]. The CLRs expressed on the surface of DCs are able to bind glycoproteins of microbial pathogens via mannose, fucose, and *N*-acetylglucosamine [47]. These CLRs can also oligomerize to facilitate enhanced ligand binding [47]. A broad division of DCs is found in peripheral blood, myeloid DCs (MDCs) and plasmacytoid DCs (pDCs) are also susceptible to infection with HIV. MDCs are more frequently found and can secrete high levels of IL-12, whereas pDCs can produce high levels of IFN-α [48]. HIV capture to DCs by binding to CD4 and CCR5 and, depending on expression of CLRs, can mediate infection through fusion with the cell membrane [40].

Following binding, activation of DCs upon HIV infection is unclear. HIV is able to escape detection from MDCs, resulting in the avoidance of viral nucleic acid coming in contact with pattern recognition receptors, which would allow MDCs to become appropriately activated. One study has found that the ability of HIV to undermine the immune system may be due to the lack of the accessory protein, vpx, in its genome. Without vpx, SAMHD1 inhibits viral replication at the level of reverse transcription, therefore enabling HIV to escape detection [49]. DCs show a resistance to HIV-1 replication from the expression of SAMHD1 [50]. In contrast, studies have shown in co-culture conditions that include both T and B-lymphocytes, this leads to a downregulation of SAMHD1 expression and is associated with increased HIV-1 replication in DCs [50]. Another study has demonstrated that PDCs exposure to gp120 interferes with TLR9 activation, decreasing its ability to secrete antiviral and inflammatory factors that play a role in initiating the immune system [51]. Overall, there has been conflicting data showing the activation of DCs upon HIV infection. Studies have shown that viral replication is inhibited, allowing HIV to escape detection, whereas other studies have shown increased viral replication stimulating an immune response.

In regards to the effects of HIV on DC maturation, studies show controversial data and results as well. It has been demonstrated that once immature DCs encounter HIV, DCs undergo maturation where molecules on the surface are upregulated and DCs can migrate from the periphery to secondary lymphoid organs [48]. Upon arrival at lymphoid tissues in mucosal transmission, HIV trans-infection to CD4+ T-follicular helper cells can occur [52]. It has been shown that DCs can undergo maturation by viral infection or by cytokines in the microenvironment during migration and then present HIV antigens to T cells in secondary lymphoid tissues and initiate an immune response [53]. Other studies have demonstrated that infection of HIV on DCs with different stimuli can lead to maturation in monocyte-derived DCs [54]. An important component of DC maturation involves DC-derived microvesicle-associated HIV particles, which are released from T lymphocytes and DCs [55]. It is well known that immature DCs are more susceptible to HIV infection in comparison to mature DCs likely due to CCR5 expression differences [56]. The presence of these microvesicles results in increased maturation of DCs, thereby reducing de novo replication of HIV [57]. Conversely, other studies have shown partial maturation of DCs that is mediated by abnormal or partial up-regulation of cell maturation markers effecting binding of gp120 to monocyte-derived DCs [58]. It has also been demonstrated that HIV infection can actively suppress DC maturation by inhibiting TLR-induced maturation of DCs [59]. The interactions between DCs is complicated and no one DC subset behaves the same. The diversity of CLRs enables DCs to broadly capture pathogens and dictate the fates of the antigen. The interactions between DCs is complicated and no one DC subset behaves the same. The diversity of CLRs enables DCs to broadly capture pathogens and dictate the fates of the antigen [60,61]. In addition to DC-SIGN, CD207, and cDC, expression of CLRs vary among DC subtypes and have been thoroughly defined [62,63]. However, further characterization of CLRs on DCs to better understand their immune function, and how they influence infection with HIV and other pathogens, may lead to novel insight to develop new strategies to prevent HIV infection of DCs.

## 7. Th1 vs. Th2 in Context of TB and HIV

In the context of *M. tb* infection, Th1 cells are hypothesized to be the critical effector subtype because *M. tb* is primarily an intracellular infection as opposed to Th2, which is typically significant in extra-cellular parasitic infections. Th1 cells clear the *M. tb* infection by their secretion of IFN-γ, which activates macrophages and CD8+ Cytotoxic T-cells (CTLs). Macrophages can then exhibit their antimicrobial properties involving phagocytosis and release of reactive nitrogen and oxygen intermediates [64]. CD8+ T-cells destroy host cells infected by intracellular *M. tb* via antimicrobial peptides, perforin, and granulysin [65].

Accordingly, patients with pulmonary TB infections have been shown to have higher Th1:Th2 ratios [66]. In TB patients treated with antimycobacterial agents, there were both a significant increase in serologic Th1 markers ((sLAG)-3) accompanied by a decrease in Th2 markers (IgE, soluble CD30, and CCL22/macrophage-derived chemokine) when compared to healthy controls at both 2–3 months and 6-month time points. Additionally, impairment of receptors or production of key cytokines, such as IFN-γ or IL-12, required for Th1 polarization and down-stream effects result in increased susceptibility to *M. tb* infection [67]. These findings indicate that the Th1 subclass plays a significant role in *M. tb* infection.

Furthermore, in vitro glutathione (GSH) treatment of immune cells isolated from *Mycobacterium bovis* Bacille Calmette-Guérin (BCG) vaccine subjects improves the immune response and clearance of the *M. tb* [65]. GSH treatment has been shown to increase production of the cytokines IFN-γ and TNF-α, resulting in enhanced effector functions of macrophages, natural killer cells, and CD8+ T-cells. GSH treatment of DCs also induces increased production of IL-12 [32]. As mentioned previously, increased IFN-γ results in an increase in IL-12 from APCs. Together, the effects of IFN-γ and IL-12 improves polarization towards the Th1 subclass and enhance the Th1 response. Overall, these studies support the importance of Th1 cells’ role in inhibiting *M. tb* infections.

Interestingly, in chronic HIV-1 infection, HIV causes the Th1 subclass to switch predominately to the Th2 subclass [68,69,70,71]. This leads to a decrease in the Th1:Th2 ratio from an alteration in cytokine balance. This is exemplified by a reduction in Th1 related cytokines, IL-2 and IFN-γ, and an increase in Th2 cytokines, IL-4 and IL-10 [70,72,73]. Furthermore, due to the down-regulation of Th1 cells and their cytokines, IL-2, IL-12, and IFN-γ, this impedes the activation of CTLs. CTLs are critical for destroying virally infected HIV cells through interactions with the MHC-I receptor. Therefore, HIV can cleverly evade its own destruction by reducing CD8+ CTL activation and response by perturbing the source of their activation, Th1 cells [74]. *M. tb* infection highly relies on the Th1 response for clearance of the infection.

In addition, HIV-1 infection and the progression to full-blown AIDS is recognized to cause a depletion in CD4+ T-cells [75]. Activated CD4+ T-cells release IL-2, a key cytokine for CD4+ and CD8+ T-cell proliferation, differentiation, and viability [76,77]. Compromised IL-2 levels prevent activation of other downstream effector cell sub-types, including CTLs, and progressive decline in CD4+ T-cell levels. Low CD4+ T-cell counts lead to increased risk for opportunistic infections, and *M. tb* is often one of the first severe opportunistic infections acquired [78]. Collectively, a shift to the Th2 subtype and decline in CD4+ T-cells overall produces an impaired ability to clear the *M. tb* infection, which is why HIV is the most significant risk factor for *M. tb* [79].

## 8. Regulatory T Cells in Context of HIV and TB

**Figure 2:**
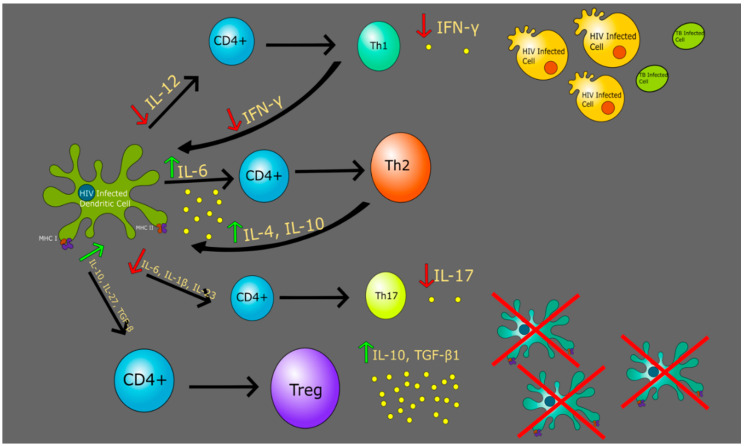
Altered immune response in an HIV-infected DC causes a decrease in cytokines such as IL-12, IL-6, IL-1β, IL-23, IL-17, and IFN-γ. The downstream effects hinder a robust immune response.

Regulatory T cells (Treg), a subset of CD4+ T cells, play a significant role in regulating the immune response as well as maintaining self-produced antigen recognition by suppression of the activation and expansion of self-reactive T cells, which prevents autoimmune disease [80]. Induction of Tregs occurs generally in central and peripheral tolerance, with those formed in the periphery being mediated by DCs (Figure 2). These peripherally formed, naturally occurring Tregs, are particularly important in maintaining homeostasis in the immune response and express CD25 and forkhead box P3 (Foxp3^+^), a transcription factor that is essential for proper Treg differentiation [36]. Of several subpopulations of Tregs with varying functions, this population of Tregs is well known and defined as CD4^+^CD25^+^FoxP3^+^ Tregs [81].

Upon formation of a granuloma, Tregs infiltrate the site of infection to regulate the increased immune response against *M. tb* and release IL-10 and TGF-β1, which suppress the actions of antigen-presenting cells like DCs [82,83]. Studies have shown an increase in levels of Tregs in patients with *M. tb* both peripherally and at the site of infection, with levels at the site of infection being greater [84,85]. The relationship of Tregs in those infected with HIV is not clearly understood as some studies have shown a deficiency of Tregs following HIV infection while others have shown an increase [64,86,87]. However, a more recent study demonstrates a decrease in Foxp3 expressing CD4 T cells during the early stages of HIV infection peripherally thus contributing to increased immune response leading to an permissive effect on HIV-1 replication [87]. Upon progression of the HIV infection, an upregulation in both levels of Tregs and Foxp3 expression with a prominent inverse relationship of CD4 count was reported, thus making these subjects more prone to being infected by active TB [87]. This suggests that the hallmark immunodeficient state in those with HIV due to loss of CD4+ cells is perpetuated by the increase in number of Tregs, further compromising the immune system and increasing the individual’s susceptibility to infection by *M. tb* [87]. However, due to the conflicting nature of data regarding the role of Tregs in HIV, further investigation is needed to clearly delineate this role in the context of HIV and TB.

## 9. Th17 Cells in Context of HIV and TB

Th17 cells, another subset of CD4+ T-cells that reside at mucosal surfaces, play a significant role in immunity and the inflammatory response, including protecting against extracellular pathogens during autoimmune diseases [88,89]. Th17 cells secrete IL-17, which serves as a chemoattractant to recruit neutrophils to the area of infection [90,91]. Differentiation, stabilization, and amplification of Th17 cells is largely dependent on the presence of TGF-β, IL-1β, IL-6, IL-21, and IL-23, although the individual roles of these cytokines are known to be different across some species, most notably between mice and humans [88].

TGF-β and IL-6 exhibit a regulatory relationship in which TGF-β influences IL-6 in its role in Th17 differentiation. Studies show that the role of TGF-β in Th17 and Treg differentiation is influenced by the inflammatory cytokine environment as levels of Foxp3^+^ were found to be inversely related to levels of IL-6, suggesting that IL-6 inhibits the generation of Tregs in the presence of TGF-β. This essentially shifts this Th17/Treg balance towards Th17 differentiation during a pro-inflammatory state [92,93]. Without Th17 inducing cytokines, high levels of TGF-β inhibit Th17 development and transitions production towards Treg cells [90]. This dynamic balance between Th17 and Tregs levels is an important part in the protection of the intestinal mucosa from pathogens [94].

HIV infection can be characterized as loss of mucosal CD4+ T cells along with a preferential loss of Th17 cells, although this mechanism is still under investigation. Despite low levels of Th17, levels of IL-6 and TGF-β have been found to be increased in those infected with HIV, therefore suggesting that this loss of Th17 cells is likely not due to a lack of Th17-inducing cytokines [95]. Th17 cells are preferentially infected in the earliest stages of transmission in cases of macaque simian immunodeficiency virus with Th17 markers CCR6 and RORγt being initial targets [96]. This permissive effect of infection on Th17 cells is likely due to multiple factors including expression of HIV dependency factors and lack of HIV restriction mechanisms. The presence of these Th17 cells on the surface of gut-associated lymphoid tissues as well as its contribution to the persistence of HIV infection, especially during antiretroviral therapy, poses Th17 cells as a potential target for immunotherapy [97]. Studies suggest that during HIV infection, Th17 cells are unable to express IL-17 and lose ability to be maintained by IL-23 [98]. This deficiency and impaired function of Th17 cells depletes the immune response at mucosal surfaces, resulting in increased translocation of microbes across the intestinal epithelium into the systemic circulation [90]. Taken into consideration in the context of *M. tb* and HIV coinfection, the respiratory mucosal defense towards inhaled *M. tb* is compromised in HIV infection largely due to this loss of Th17 cells. In conjunction with reduced IL-17 chemoattractant properties, as well as an increase in the number of Tregs resulting in immunosuppression, this results in advancement of active TB infection [95].

## 10. Glutathione with Dendritic Cells to Improve Immune Response

Studies have shown that redox homeostasis can regulate the functions of DCs against both TB and HIV infections. Glutathione (γ-l-glutamyl-l-cysteinyl-glycine) is a tripeptide antioxidant composed of glutamine, cystine, and glycine. It is found at millimolar concentration in nearly all eukaryotic as well as many prokaryotic cells [99,100]. The synthesis of glutathione is driven by system x_c_^−^ (Sx_c_^−^), an amino acid antiporter that mediates the exchange of extracellular l-cystine (L-Cys_2_) and intracellular l-glutamate (L-Glu) across the cellular plasma membrane. The influx of L-Cys_2_ is the rate-limiting step in providing the intracellular L-cysteine required for the synthesis of GSH [101]. In multiple cell types, GSH plays critical roles in protecting cells from oxidative damage and maintaining redox homeostasis. The redox cycle consists of the balance of reduced (GSH) and oxidized (GSSG) glutathione [102]. When exposed to reactive oxygen species (ROS), two molecules of GSH are converted to GSSG and water. In this mechanism, GSH contains antioxidant properties, whereas GSSG is a simple by product of the oxidation of GSH with no antioxidant effects.

**Figure 3:**
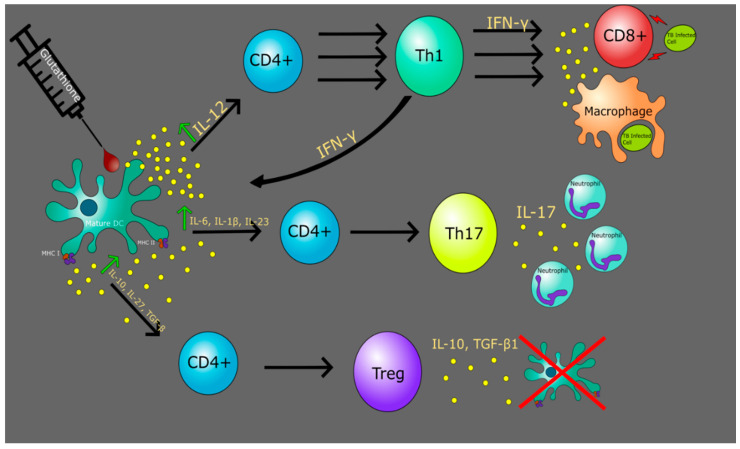
The addition of the antioxidant glutathione maintains redox homeostasis of DCs. This leads to an enhanced response from DCs in cytokine production, causing a robust immune response to infections.

GSH plays a critical role in maintaining DC redox homeostasis (Figure 3). Stimulation of DCs by T cells activates the nuclear factor kappa-light-chain-enhancer of activated B cells (NF-κB) pathway in DCs and induces an antioxidant response. It also enhances system x_c_^−^-dependent cystine uptake, leading to increased GSH synthesis, export, and finally, degradation to cysteine outside the cell [103]. Studies have shown that an altered cellular redox has a profound role in inflammation through the activation of various kinases and redox-sensitive transcription factors such as NF-kB rel proteins, which differentially regulate the genes encoding various pro-inflammatory cytokines [104]. Severe GSH depletion impairs the DCs ability to reduce antigen disulfide bonds required before antigen processing [105] and decreases the activity of thiol proteases important in antigen processing and cleaving of the invariant chain from MHC II [106]. The altered T-cell responses to antigen presentation presented by GSH-depleted DCs reflect changes in the production of IL-12. IL-12 is produced by the activation of inflammatory cells, such as DCs, macrophages, and monocytes and is a key regulator of T-cell differentiation via the formation of IFN-γ [107]. The addition of exogenous GSH to DCs results in the recovery of TNF-α, IL-6, and IL-12 [108]. The dramatic changes presented by GSH depletion are consistent with the fundamental roles GSH plays in cellular physiology and may play a key role in developmental medications for various infections.

## 11. The Role of GSH in Altering TB and HIV DC Functionality

*M. tb* infects DCs via ligation of DC-SIGN by the mycobacterial surface-exposed lipoglycan lipoarabinomannan (LAM) [109]. By increasing intracellular levels of GSH, thereby stimulating the NF-kB rel proteins, DC performance is enhanced in its innate function by inhibiting the intracellular growth of *M. tb* as well as its adaptive immune role as the key APC [32]. In an *M. tb* infection, exogenous GSH addition results in more robust granuloma formation due to the enhancement of DC and cytokine activity. Further research is needed to determine the mechanism by which DC enhanced functionality against *M. tb* infection occurs.

Cystine, the rate-limiting factor in GSH biosynthesis, is supplied to lymphocytes during their encounter with DCs [110], and, upon activation, is necessary for proliferation of T lymphocytes for their G_1_/S phase [111]. The presence of gp120 found on the surface of HIV suppresses the expression of GSH synthesizing and metabolizing enzymes [112]. This is further evidenced with diminished levels of GSH in plasma, erythrocytes, and peripheral blood mononuclear cells (PBMCs) of HIV+ individuals. This reduction was coupled with decreased levels of cystine in plasma, thereby affecting DC functionality [111,113]. The net result led to oxidative stress linked with increased viral replication [114]. The addition of N-acetylcysteine (NAC), a prodrug that supplies bioavailable cysteine, replenishes whole blood GSH and T cell GSH, offering useful adjunct therapy to increase protection against oxidative stress [115].

## 12. Summary

Research has shown that DCs play an important role in mucosal immunity. Given the mode of transmission for HIV via mucosal transmission, the first cells infected are the DCs by the dendrites on the cell surface. If a treatment strategy is formed to target dendrites, this could be a breakthrough for HIV infection. Previous studies have demonstrated that the loss of DCs could be relevant in treatment strategies for TB and HIV. Immunotherapies and vaccines specific for strengthening DCs could result in further management of these diseases. However, these treatments cannot be accomplished with the lack of research. Knowledge gaps still persist in the class of DC that should be targeted to reduce further loss in response to an HIV or TB infection. Further research in the field of GSH enhancing DCs could lead to the beginnings of immunotherapy for these immunocompromised patients.
